# Identifying more reliable parameters for the detection of change during the follow-up of mild to moderate keratoconus patients

**DOI:** 10.1186/s40662-017-0089-3

**Published:** 2017-10-25

**Authors:** Ivo Guber, Colm McAlinden, François Majo, Ciara Bergin

**Affiliations:** 1 Department of Ophthalmology, University of Geneva, Geneva, Switzerland; 2 Department of Ophthalmology, Glangwili Hospital, Hywel Dda University Health Board, Carmarthen, UK; 3 School of Ophthalmology and Optometry, Wenzhou Medical University, Wenzhou, Zhejiang China; 4 Jules-Gonin Eye Hospital, University of Lausanne, Fondation Asile des Aveugles, Lausanne, Switzerland

**Keywords:** Pentacam, Keratoconus, Progression, Repeatability, Precision, Corneal crosslinking

## Abstract

**Background:**

Reaching a consensus on which parameters are most reliable at detecting progressive keratoconus patients with serial topography imaging is not evident. The aim of the study was to isolate the parameters best positioned to detect keratoconus progression using the Pentacam HR® measures based on the respective limits of repeatability and range of measurement.

**Method:**

Using the Pentacam HR®, a tolerance index was calculated on anterior segment parameters in healthy and keratoconic eyes. The tolerance index provides a scale from least to most affected parameters in terms of measurement noise relative to that observed in healthy eyes. Then, based on the “number of increments” from no disease to advanced disease, a relative utility (RU) score was also calculated. RU values close to 1 indicate parameters best positioned to detect a change in keratoconic eyes.

**Results:**

The tolerance index values indicated that 36% of ocular parameters for keratoconic eyes had repeatability limits which were wider than normative limits (worse), but 28% of the ocular parameters were narrower than normative limits (better). Considering only those parameters with a RU greater than 0.95, a small number of parameters were within this range, such as corneal curvature and asphericity indices.

**Conclusions:**

This study demonstrates that measurement error in keratoconic eyes is significantly greater than healthy eyes. Indices implemented here provide guidance on the levels of expected precision in keratoconic eyes relative to healthy eyes to aid clinicians in distinguishing real change from noise. Importantly maximal keratometry (Kmax), central corneal thickness (CCT) and thinnest corneal thickness (TCT) were highlighted as problematic indices for the follow-up of keratoconus in terms of repeatability.

## Background

The clinician who follows keratoconus patients with serial topography imaging desires to know which parameters are most reliable at detecting progression. This is important as the detection of progression will often determine treatment choice e.g., collagen cross-linking (CXL). However, the ability of a parameter to detect progression is decreased with increased measurement noise (signal to noise ratio). Previously, in healthy eyes the repeatability limit, of the maximal corneal curvature Kmax (with the Pentacam HR®) were reported to be 0.8 Dioptres (D), however, we found repeatability limits in keratoconic eyes to be 1.97 D [1, 2]. This result means that the current main criteria for progression detection and CXL is inadequate (i.e. a change of 1 D in Kmax after one year of follow-up) [1, 3].

To date, comparison of repeatability between subgroups has been limited to a comparison of the repeatability limits or the correlation of variation values within a given parameter [4–16]. Noting the important changes observed in repeatability limits with keratoconus, we aimed to determine which parameters were least affected. To isolate these parameters, we employed the tolerance and relative utility (RU) indices [17]. The tolerance index creates a scale of least to most affected parameters and the RU index highlights which parameters will theoretically describe the most number of stages of severity of disease and hence progression.

In this article, we aim to highlight the topographic parameters obtained with the Pentacam HR® (V 1.20r02) that are more reliable in detecting keratoconus progression. We aim to achieve this by providing a table of the associated tolerance and RU indices and demonstrating their use.

## Methods

This study was approved by the local cantonal ethics committee and adhered to the tenets of Declaration of Helsinki for research on human subjects. Informed consent was obtained from all participants.

### Patients

#### Healthy group

Ethical approval was granted by the Flinders clinical research ethics committee. Data from a previous study by McAlinden et al. were used as the healthy control group [2]. This study reported the repeatability limits of Pentacam HR parameters for 100 healthy eyes. These reported repeatability limits were used to calculate the tolerance index and RU index. The study by McAlinden et al. involved the use of one randomly selected eye. For the repeatability assessment, each eye was scanned twice with the Pentacam HR in the 25 pictures per second mode using automatic release by one observer. Participants remained positioned during all repeated measurements. Only scans that had an examination quality specification graded as “OK” were saved. Fifty-three left eyes of 100 subjects (68 female) with a mean age of 33.7 years (range 19–68) were included. A sample size of 100 eyes will give 99% confidence limits around estimates that are within 13% of the true value. McAlinden et al. reported an estimate of 95% limit of repeatability in K-max to be 0.8 D in normal subjects, therefore the 99% confidence interval (CI) around the estimate of the 95% limit is 0.7 D and 0.9 D.

#### Keratoconus (KCN) group

Ethical approval was granted by the ethical commission of the canton de Vaud, Switzerland under protocol number 375/11. Thirty-three eyes of 20 patients with mild to moderate KCN were recruited from a specialized anterior segment unit at the Jules-Gonin eye hospital in Lausanne, Switzerland. Tomography measurements were obtained using the Pentacam HR® (V 1.20r02). Three repeated measurements by two independent observers were taken with the Pentacam HR in the 25 pictures per second scanning automatic release mode by two independent observers. Only measurements with a quality factor (Q) “OK” or when over 95% of the data was validated by the system were used for analysis. Images from 32 eyes (16 right, 16 left) of 20 patients (6 females, 14 males) were taken. The mean age of patients was 31 years (range 18–47). Baseline mean and standard deviation (SD) for thinnest corneal thickness (TCT), maximal corneal curvature (Kmax), mean corneal curvature anterior (Km ant), astigmatism, anterior chamber (AC) depth and corneal volume (CV) at 7 mm were 482.1 ± 36.8 μm, 52.3 ± 3.7 D, 46.0 ± 2.2 D, −3.25 ± 1.6 D, 3.3 ± 0.3 mm, and 23.5 ± 1.6 mm^3^, respectively. A sample size of 32 will give 99% confidence limits that are within 23% of the true value; here we have reported that K max has a repeatability of 1.97 D, therefore the 99% CI of this estimate is 1.5 D and 2.4 D.

### Tolerance index

Repeatability (*Sr*) and reproducibility (*SR*) were assessed based on the recommendations from the British Standards Institute and the International Organization for Standardization [18]. Repeatability and reproducibility limits from the normal population are denoted as r_N_ and R_N_ [2]. Repeatability and reproducibility limits derived from our KCN population are denoted as r_K_ and R_K_ [1]. These were used to calculate the tolerance index, denoted as Tr and TR for repeatability and reproducibility limits, respectively [17].

1 $$ {Tr}_i={\mathrm{Log}}_n\left(\frac{r_{K_i}}{r_{N_i}}\right);\kern0.5em {TR}_i={\mathrm{Log}}_n\left(\frac{R_{K_i}}{R_{N_i}}\right) $$

Where i represents the *i* ^th^ parameter e.g., Kmax, K1 etc. A tolerance index value of 0 represents perfect agreement with normal limits; the larger the difference from 0 the greater divergence from normative limits. Negative numbers indicate narrower (better) CI limits in the pathological group relative to normal subjects and positive numbers indicate wider (worse) CI limits.

### Sample size

Based on the estimates of repeatability of each parameter (e.g., K-max) in both populations, healthy (*n* = 100) and keratoconic (*n* = 32), the respective CI around each estimate can be calculated and CI overlap can be assessed. In this way, any significant changes in repeatability can be detected and highlighted. The tolerance index allows us to summarize this information systematically. Based on the central limit theorem, with a sample size of 32 and 100, a │tolerance value│of >0.24 indicates that the confidence limits do not overlap and there is a statistically significant difference at the 5% level.

### Relative utility index

To derive the RU, the within-subject standard deviation for repeated measures that is derived by a one-way analysis of variance (ANOVA)(*Sr*_*i*_), the between observer standard deviation that is derived by ANOVA (*SR*_*i*_), and the between patient standard deviation (*SP*_*i*_) were calculated using the data in keratoconus eyes (Eq. 2).

2 $$ {RU}_i=\sqrt{\frac{SP_i^2}{SP_i^2+{SR}_i^2+{Sr}_i^2}} $$

The RU scale is from 0 to 1, with poor latent ability nearer 0 and good latent ability nearer to 1. Analysis was performed with R software version 2.15.1 [19].

## Results

### Repeatability and tolerance index

The tolerance index values reported for anterior and posterior curvatures were on average greater than +0.35, in particular, Kmax had a Tr of 0.90 indicating a much wider repeatability limit in keratoconus eyes compared to normal eyes (Table 1). On the other hand, anterior and posterior axis values were found to demonstrate better repeatability limits (*r* = 11°; 23° respectively), with better (high negative) *Tr* values (*Tr* < −1.7). Summary data in terms of keratometric power deviation (KPD), AC depth, AC volume and AC angle estimates were all greater than normative values (Table 1; *Tr* > 0). Front surface elevation maps at TCT were more repeatable than back surface elevation maps at TCT. Pachymetry estimates had good repeatability limits for pupil centre, corneal apex, and TCT, with most measures inside normal limits (Table 1). Corneal volume measurements at all diameters were repeatable and had similar or better than normative limits of repeatability (*Tr* < 0.2). The topometric *Q-*values were repeatable, however, anterior *Q*-value repeatability limits were outside normal limits. Centre keratoconus index (CKI) and index of height decentration (IHD) were repeatable with tighter limits of repeatability (*Tr* < −1.1) but index of surface variance (ISV), index of vertical asymmetry (IVA) and particularly index of height asymmetry (IHA) were markedly less repeatable and significantly outside normative limits (*Tr* > 1.0).

**Table 1:** The tolerance indices (Tr, TR)

	Repeatability	Reproducibility		
		Single	Pair	Triplet
Anterior	Tr	TR	TR	TR
K1 (D)	−0.12	−0.43	−2.00	−4.33
K2 (D)	−0.08	0.12	−3.39	−0.57
Km (D)	0.16	0.08	−1.59	−0.23
Axis (deg)	−2.17	−2.84	−2.90	−4.39
Q-value, 30 deg	0.73	0.78	0.62	0.89
Rper (mm)	1.02	0.43	1.49	1.16
Rmin (mm)	0.33	0.52	−0.02	−0.23
Posterior				
K1 (D)	0.89	0.51	1.03	1.26
K2 (D)	0.92	0.89	−1.77	0.39
Km (D)	0.33	0.59	0.79	1.06
Q-value, 30 deg				
Rper (mm)	0.55	0.24	0.29	−0.83
Rmin (mm)	0.73	0.90	1.33	0.57
Summary data				
Kmax front (D)	0.90	1.06	0.50	0.12
X-axis (mm)	−1.79	−1.03	−0.90	−0.93
Y-axis (mm)	−1.78	−2.15	−3.54	−2.43
Corneal volume (mm^3^)	0.13	0.86	0.58	0.12
KPD (D)	0.58	0.52	−0.16	−0.86
AC volume (mm^3^)	0.80	−0.11	−0.84	−0.81
AC angle (deg)	0.25	0.60	−0.08	−1.99
AC depth (mm)	1.31	0.35	−1.26	−0.60
Pupil diameter (mm)	−0.22	−0.77	−0.11	−0.19
Pachymetry				
Pupil centre (μm)	−1.98	−1.45	−1.99	−2.21
X-axis (mm)	−4.61	−5.32	−8.32	−7.48
Y-axis (mm)	−3.96	−6.12	−6.16	−4.91
Apex (μm)	0.17	1.05	0.70	0.24
TCT (μm)	−0.42	1.09	0.92	0.77
Corneal volume (mm^3^)				
3 mm diameter	−2.31	−0.60	−0.56	−0.78
5 mm diameter	−1.55	−0.75	−1.01	−1.32
7 mm diameter	−0.25	0.85	0.59	0.19
Indices				
ISV	1.04	−0.32	−1.11	0.22
IVA	1.02	1.35	0.88	−0.38
KI	−0.08	0.92	0.72	0.52
CKI	−0.08	−1.61	−2.51	−2.76
IHA	2.09	2.41	2.49	2.43
IHD	−1.10	−1.66	−0.57	−0.73

*K1, K2* = Keratometry readings 1,2; *Km* = Mean keratometry reading; *Rper* = Mean radius of curvature in the 7-9 mm area of the cornea; *Rmin* = Minimum radius of curvature; *KPD* = Keratometric power deviation; *AC* = Anterior chamber; *ISV* = Index of surface variance; *IVA* = Index of vertical asymmetry; *KI* = Keratoconus index; *CKI* = Centre keratoconus index; *IHA* = Index of height asymmetry; *IHD* = Index of height decentration; *Sr* = Within-subject standard deviation; *r* = The limits of repeatability; *Tr* = The log of the ratio between the limits of repeatability of keratoconus patients and normal subjects; −0.45 < Tr/TR < 0.45 is within normative limits

Example of reading the table: Taking the line Kmax, r is 1.97 D, therefore it has a Tr value of 0.9, which means that r limit of 1.97 is well outside normal limits, R is 2.3 D, therefore it has a TR value of 1.06 is reported when a single image is used, again indicating that this is well outside normal limits, TR reduces to 0.12 when the average of three images was used, which is not significantly different than normal limits

### Reproducibility and tolerance index

With a single image, Kmax had reproducibility limits well outside normal with a *TR* value of 1.06, but when the average of three images was used instead, reproducibility was similar to normal limits (*TR* = 0.12). Of the pachymetry estimates, apex measures were the least reproducible followed by those at the TCT. The measures at pupil centre had the best R-value (R-values, Table 1). R-values of corneal volume increased with increasing diameter, however central corneal volume R-limits were greater than any of the peripheral estimates. Anterior *Q*-values had worse reproducibility than normal limits and did not markedly improve when estimates from pairs or triplets of images were used. IHD and CKI had tight reproducibility limits, remaining within normative limits, suggesting these are among the most reproducible parameters in KCN patients.

### Relative utility index

RU was used to indicate which parameters are less variable relative to the respective dynamic range of that parameter in our cohort (Table 2). Pachymetry at the corneal apex, for example, is unlikely to be useful clinically, as this parameter has an RU of 0.42, suggesting that 58% of the differences in CT apex between any two keratoconic eyes from the study cohort can be attributed to measurement variability (Table 2). On the other hand, corneal curvature estimates all have RU values above 0.94, except for Kmax that has an RU of 0.88 (Table 2). Considering only those parameters with a RU value greater than 0.95, a small number of parameters within the acceptable range were identified, namely: K1, K2 and Km; *Q*-value (anterior), R-peripheral posterior, CKI, ISV, IVA, IHD, AC depth, the back-elevation map at TCT and ectasia map indices D and Db (Table 2).

**Table 2:** Summary of variation between patients and ratio of variability attributable to instrument and observers

	SP	S(r&R)	r&R	TV	RU
Anterior					
K1 (D)	4.14	0.10	0.28	4.14	1.00
K2 (D)	3.11	0.19	0.53	3.11	0.99
Km (D)	3.66	0.19	0.53	3.66	0.99
Astigmatism (D)	1.02	0.16	0.46	1.04	0.91
Q-value, 30 deg	0.83	0.08	0.24	0.84	0.98
Rper (mm)	0.07	0.04	0.10	0.08	0.76
Rmin (mm)	0.52	0.11	0.32	0.53	0.90
Posterior					
K1 (D)	1.06	0.09	0.24	1.07	0.99
K2 (D)	0.78	0.14	0.39	0.79	0.94
Km (D)	0.96	0.06	0.18	0.96	0.99
Astigmatism (D)	0.24	0.09	0.25	0.26	0.68
Q-value, 30 deg	0.82	0.19	0.54	0.84	0.85
Rper (mm)	0.42	0.06	0.18	0.42	0.97
Rmin (mm)	0.48	0.09	0.26	0.49	0.92
Summary data					
Kmax front (D)	4.56	1.09	3.03	4.69	0.88
Corneal volume (mm^3^)	4.39	1.34	3.72	4.59	0.79
KPD (D)	0.47	0.15	0.41	0.49	0.83
AC volume (mm^3^)	48.84	14.65	40.60	50.99	0.92
AC angle (deg)	15.47	4.91	13.60	16.23	0.80
AC depth (mm)	0.72	0.04	0.12	0.72	1.00
Pupil diameter (mm)	0.46	0.18	0.51	0.49	0.84
Pachymetry					
Pupil centre (μm)	28.20	11.00	30.49	30.27	0.72
Apex (μm)	13.76	11.54	31.98	17.96	0.42
Thinnest (μm)	10.28	9.84	27.28	14.24	0.36
Corneal volume (mm^3^)					
3 mm diameter	0.05	0.06	0.17	0.08	0.34
5 mm diameter	0.14	0.14	0.40	0.20	0.35
7 mm diameter	0.85	0.44	1.23	0.96	0.59
Indices					
ISV	42.03	3.12	8.64	42.15	1.00
IVA	0.68	0.05	0.14	0.68	0.98
KI	0.13	0.03	0.09	0.14	0.85
CKI	0.05	0.01	0.03	0.05	0.96
IHA	17.95	18.40	51.01	25.71	0.39
IHD	0.04	0.00	0.00	0.04	0.96
Elevation map					
Front at TCT (μm)	18.33	4.82	13.36	18.95	0.85
Back at TCT (μm)	55.01	4.64	12.85	55.21	0.99
Ectasia map					
Df	6.41	2.30	6.38	6.81	0.75
Db	5.57	0.77	2.14	5.62	0.99
Dp	2.94	1.45	4.01	3.28	0.60
Dt	0.12	0.13	0.37	0.18	0.39
Da	0.10	0.18	0.51	0.21	0.21
D	4.13	0.46	1.28	4.16	0.99

*K1, K2* = Keratometry readings 1,2; *Km* = Mean keratometry reading; *Rper* = Mean radius of curvature in the 7-9 mm area of the cornea; *Rmin* = Minimum radius of curvature; *KPD* = Keratometric power deviation; *AC* = Anterior chamber; *ISV* = Index of surface variance; *IVA* = Index of vertical asymmetry; *KI* = Keratoconus index; *CKI* = Centre keratoconus index; *IHA* = Index of height asymmetry; *IHD* = Index of height decentration; *SR* = Between observer standard deviation; *R* = The limits of reproducibility; *TR* = The log of the ratio between the limits of reproducibility of keratoconus patients and normal subjects; *SP* = Between patient standard deviation; *S(r&R)* $=\sqrt{Sr^2+{SR}^2}$; *TV* = Total variation $=\sqrt{SP^2+{Sr}^2+{SR}^2}$; *Df* = Deviation of the front elevation map; *Db* = Deviation of the back elevation map; *Dp* = Deviation of average pachymetric progression; *Dt* = Deviation of the minimum thickness; *Da* = Deviation of the apex thickness; *D* = Belin/Ambrosio ectasia total deviation value

Example of reading the table: Kmax front has an SP value of 4.56 D, which represents the average between patient variability in this parameter; S(r&R) represents the combined limits of repeatability and reproducibility: 1.03 D, TV of 4.78 D represents the total in Kmax variability due to repeatability, reproducibility and between-patient variability. The associated RU value of 0.88 indicates that this parameter has considerable noise with respect to the range of the dynamic range

## Discussion

Clinically, it is difficult to choose which parameter to use to determine whether disease progression has occurred, a consensus on the accepted parameters is emerging but there is still significant divergence between authors [1–18, 20–26]. This article provides an overview of the reliability of these parameters, removing the clinical interpretation component. We have summarized the differences in measurement noise between healthy and keratoconus patients across all topographic parameters from the Pentacam HR device using the *tolerance index*. Comparing “r” and “R” reported by McAlinden et al. in healthy eyes to our data in keratoconic eyes, 36%/44% (*n* = 13/36; 16/36) of parameters were significantly worse (*Tr*/*TR* > 0.45), and 28%/36% (*n* = 11/36; 13/36) were significantly better (*Tr*/*TR* < −0.45) (e.g., axis is more repeatable in KCN patients) [1, 2].

Furthermore, our study data demonstrates that averaging across several images significantly improves the tolerance values, or results in lower level of measurement noise; some parameters recovering to those levels observed in healthy eyes [20]. For example, using the average of three images instead of a single image reduced reproducibility limits of Kmax to be in line with normal values (Table 1). These results indicate that if the average of three topographies instead of a single topography was automatically calculated, the ability to detect keratoconus progression could be significantly improved.

Using this information, the RU index isolated the group of parameters theoretically best positioned to detect progression. Summarizing the RU values: 37% (*n* = 15/41) of parameters had an RU greater than 0.95, indicating good ability to detect progression, 29% (*n* = 12/41) of parameters had an RU <0.80 indicating poor ability to detect progression. It may seem counterintuitive, but it is possible that a parameter has poor TI but still a good RU. This is because some parameters have large differences between mild and moderate KCN or in other words has a large dynamic range, and it is the balance between the limits of repeatability and the size dynamic range that determines the RU.

Clinically, there are three primary motivations for collecting serial topography images in keratoconus patients: to help distinguish healthy from early keratoconus, to detect progression of keratoconus, or to determine the effectiveness of treatments for keratoconus. Regardless of the motivation, when comparing the RU values reported in this article with the area under the curve (AUC) values reported in the literature, we observe that there is notable agreement [4, 5, 7–15, 24].

In studies which attempt to distinguish between healthy and keratoconic eyes, the pachymetry values, posterior elevations maps, keratometry asymmetry and decentration indices have been mainly reported [4, 5, 7–10, 13, 14]. Pachymetry at centre and thinnest location have good sensitivity and specificity, however, the AUC is lower than that reported with the asymmetry indices [7, 8, 25]. Comparing the parameters with >0.90 AUC values reported by Correia et al. to those parameters with >0.95 RU values reported here, there is good agreement [7]. Likewise, comparing the poorest AUC results (<0.85) reported by Uçakhan et al. to the poorest RU values (<0.8) reported here, there is good agreement in majority of parameters [8].

There are several articles examining keratoconus progression [5, 11, 12, 15]. The corneal curvature parameters perform well in distinguishing between different stages of the disease [5], furthermore progressing eyes have significantly different change rates in these parameters than in non-progressing eyes [15], which corresponds well with RU values recorded here for K1, K2 and Km. Despite central corneal thickness (CCT) and TCT being well established clinically and both demonstrating significant difference in mean values for different stages of the disease [11], the annual change rates are not significantly different between progressing and stable eyes for these parameters [15], which corresponds to the poor RU values for pachymetry reported in this study (RU < 0.75).

There are a small number of studies that have examined topographic parameters following CXL: those parameters with positive outcomes in these studies correspond well with the better RU values reported in this study [22–24]. In our study, the large change in repeatability in eyes with keratoconus versus healthy eyes indicates that repeatability in eyes following CXL should be critically examined, as there are many possible additional confounders. A change in repeatability in eyes following CXL could be important, as currently there is more than 70 clinical trials listed on the National Institute for Health Research (NIHR) clinical trial registry examining the effectiveness of CXL, where the primary or secondary outcome is a change in corneal curvature. Therefore, the parameters used to validate keratoconus progression in these clinical trials may require updating.

This agreement between RU and AUC values is of significance as the data required to calculate RU values is collected at one sole visit, while the AUC data requires data from several years of clinical observation. RU values are not a replacement for AUC values, but they can be used to help optimize clinical trials, by helping to provide guidelines on the parameters of interest, the optimal number of scans and the frequency of consultation.

Some of the differences in precision noted between keratoconic and normal eyes are likely to be related to the fitting algorithm used by the Pentacam HR device. Alignment algorithms rely on alignment markers such as pupil centre, thinnest corneal location, and corneal apex. Some alignment markers will be less evident in normal eyes than keratoconic eyes. For example, due to the conical shape of the cornea in keratoconic eyes, the location of Kmax is clear in most images, therefore the same x, y coordinates will be calculated between images. Furthermore, the fitting algorithm uses a model of the smooth spherical cornea in the form of a “best fit sphere” more akin to the normal cornea than the conical cornea observed in keratoconic eyes. With this technique, the presence of the cone is unexpected and likely distorts estimates of many of the topographic parameters [6]. Lastly, in eyes with a steep cone, the eye movements associated with the loss of fixation have the potential to cause much larger errors in the estimation of parameters such as Kmax and TCT. This may be exacerbated by multifocality associated with these “steep cones”, thus greater higher intraocular straylight [16, 21], and poorer fixation. Lastly, this study examined only early to moderate KCN, those parameters identified as useful in this group may differ from those used in more advanced disease [25–27].

## Conclusion

The indices implemented in this article were designed to provide an “at a glance” guideline on the levels of expected precision in keratoconic eyes relative to healthy eyes to aid clinicians in distinguishing real change from variability [18]. Furthermore, the RU index isolates topographic parameters with a large dynamic range in comparison to measurement noise. This index gives an indication of those parameters with the potential for detecting a change when no longitudinal data are available e.g. when a new device/software is released. Our hypothesis is that parameters with high RU are best positioned to detect change, whether it is disease progression or assessing the efficacy of a therapeutic intervention. For example, the Kmax and CCT parameters, which are currently the standard measures used for the monitoring of keratoconus have been shown to have poor RU in our study, indicating that these parameters are not best positioned to detect change. Further investigation is required to verify these results and develop this methodology for clinical practice.

## Abbreviations

AC: Anterior chamber
AUC: Area under the curve
CKI: Centre keratoconus index
CXL: Collagen cross-linking
D: Belin/Ambrosio ectasia total deviation value
D: Dioptre
Da: Deviation of the apex thickness
Db: Deviation of the back-elevation map
Df: Deviation of the front elevation map
Dp: Deviation of average pachymetric progression
Dt: Deviation of the minimum thickness
IHA: Index of height asymmetry
IHD: Index of height decentration
ISV: Index of surface variance
IVA: Index of vertical asymmetry
K1, K2: Keratometry readings 1 and 2
KCN: Keratoconus
KI: Keratoconus index
Km: Mean central keratometry
KPD: Keratometric power deviation
r: Limits of repeatability
R: Limits of reproducibility
Rmin: Minimum radius of curvature
Rper: Mean radius of curvature in the 7-9 mm area of the cornea
RU: Relative utility
Sr: Repeatability
SR: Reproducibility
TR: Tolerance index (the log of the ratio between the limits of reproducibility of keratoconus patients and normal subjects)
